# Health Related Quality of Life in Iranian Hemodialysis Patients With Viral Hepatitis: Changing Epidemiology

**DOI:** 10.5812/hepatmon.9611

**Published:** 2013-05-30

**Authors:** Zohreh Rostami, Mahboob Lessan Pezeshki, Azam Soleimani Najaf Abadi, Behzad Einollahi

**Affiliations:** 1 Nephrology and Urology Research Center, Baqiyatallah University of Medical Sciences, Tehran, IR Iran; 2 Department of Nephrology, Tehran University of Medical Sciences, Tehran, IR Iran; 3 Department of Nephrology, Artesh University of Medical Sciences, Tehran, IR Iran

**Keywords:** Viral Hepatitis, Hemodialysis, Hepatitis B, Hepatitis C

## Abstract

**Background:**

There are surprisingly a few studies that evaluate the impact of chronic viral hepatitis, which is common in HD (hemodialysis) patients, on HRQOL (health related quality of life).

**Objectives:**

We conducted a study to evaluate the impact of chronic viral hepatitis on HRQOL and to compare their HRQOL with non-infected HD patients via a HRQOL questionnaire.

**Patients and Methods:**

The Iranian adapted version of the Kidney Disease Quality of Life Short Form (KDQOL-SF) version 1.3 questionnaires were filled out by the HD patients. In all HD patients, serum HBsAg, HBS Abs, and HCV Abs [enzyme-linked immunosorbant assay (ELISA)] were routinely checked every six months. Patients were considered to have chronic HBV infection if HBsAg was positive for more than six months. In all HD patients, third generation assay was used to detect HCV infection. Furthermore, serum HCV-RNA (PCR) was examined in anti-HCV-positive patients for confirmation of HCV infection.

**Results:**

in this cross sectional study 4101 patients from 103 dialysis units in Iran between October 2010 and August 2011 were included. Prevalence of hepatitis B and hepatitis C infection was 2.1% and 1.3% respectively. Almost all KDQOL items for viral hepatitis patients had equivalent or better scores than those without viral hepatitis. In the logistic regression after adjustment for age, sex, educational level, marital status, dialysis vintage, HBs Ag positivity and HCV Ab positivity, only age (P < 0.001) and educational level (P = 0.015) had negative impact on quality of life.

**Conclusions:**

Our data show that not only general health and physical activity were preserved but also health perception may be better among HD patients with viral hepatitis.

## 1. Background

Viral hepatitis in hemodialysis (HD) patients is more prevalent than the general population (1, 2). In addition, recent evidence has emphasized the adverse effects of HBV and/or HCV infection on survival and certainly on health related quality of life (HRQOL) in this population (3). Several reports have revealed that both end stage renal disease (ESRD) and viral hepatitis have negative impact on HRQOL, mortality and morbidity (1, 3). Nevertheless, the mechanisms underlying impairment in HRQOL have not been fully elicited (1, 4, 5). Although HRQOL in asymptomatic HBV carriers with normal renal function is as good as those with HBsAg negative; progression of disease worsens the general health and consequently the mental health. Finally, when it progresses to ESRD all dimensions of HRQOL are influenced (6). Moreover, HRQOL in HCV-infected HD patients is correlated with anemia, malnutrition, and depression, and is not associated with severity of the liver disease (5). The natural history of HBV and HCV infections in patients with ESRD are not entirely implicated (3) and the prevalence of HCV and HBV infection in HD patients differs noticeably country-by-country, ranging from negligible for HBV infection in developed countries to a very high prevalence of HCV infection in some developing countries (7, 8). As prevalence of hepatitis virus infections is higher in developing countries than in developed countries (9), due to accessibility of healthcare facilities, the factors that inﬂuence HRQOL in patients with ESRD especially in association with other comorbid diseases would undoubtedly be variable. Although, the trend of the interaction between multiple diseases and HRQOL has steadily increased, previous researches have focused on the effects of erythropoietin and exercise, and other factors on HRQOL among dialysis patients (10-12). Despite increasing interest in assessing HRQL in different types of chronic disease, there are surprisingly a few studies that evaluate the impact of chronic viral hepatitis, which is common in HD patients (13), on HRQOL (14).

## 2. Objectives

We conducted a study to evaluate the impact of chronic viral hepatitis on HRQOL and to compare their HRQOL with non-infected HD patients via a HRQOL questionnaire.

## 3. Patients and Methods

### 3.1. Patients

In a prospective cross-sectional study, the quality of life was evaluated among 4101 adult HD patients who were checked for hepatitis B surface antigen (HBsAg) and hepatitis C antibody (HCV Ab) from 103 dialysis units in Iran between October 2010 and August 2011. The Iranian adapted version of the Kidney Disease Quality of Life Short Form (KDQOL-SF) version 1.3 questionnaires (15) was filled out by these HD patients. We enrolled patients aged more than 14 years that were clinically in a stable condition, who remained on HD for at least 3 months and received HD 3 times per week with each session lasting 3 to 4 hours. We excluded the patients hospitalized for an acute illness, those with vascular access failure, including dialysis via a temporary vascular access and those who refused to participate in the study. All patients were divided into three groups; group I, patients who were serologically negative for HBsAg and HCV Ab (n = 3961); group II, patients with HBsAg positive (n = 87); and group III, patients with HCV Ab positive (n = 53).

### 3.2. Instruments

The evaluation of HRQOL was perform by the KDQOL-SF questionnaire version 1.3 ( 16 ), which includes generic and disease specific cores. Mental and Physical Component Summary, (MCS and PCS) was obtained from the generic core scores, that encompass eight scales of the SF-36 physical functionality , role-physical, bodily pain, general health, vitality (energy/fatigue), social function, mental health (emotional well-being), and role-emotion. Disease specific items were condensed in to eleven scales targeted for kidney disease and including symptoms/problems, effects of kidney disease on daily life, burden of kidney disease, work status, cognitive function, quality of social interaction, sexual function, sleep, social support, dialysis staff encouragement and patient satisfaction. These 11 kidney disease specific scales of the KDQOL-SF questionnaire were validated for Iranian patients ( 15 ) and were condensed as the Kidney Disease Component Summary (KDCS). Each scale score varied from 0 to 100 points; hence, higher scores indicated a better quality of life. Details on translation and validation of SF-36 Health Survey have been described elsewhere ( 17 ); however, KDQOL-SF version 1.3 underwent a translation and validation process to Farsi in another study ( 15 ). In our study, all scales had internal consistency and reliability as measured according to Cronbach alphas ranging from 0.41 to 0.91 (Table 1). The questionnaire was generally self-administered, where the respondent completed it on his/her own; therefore, the patients mostly filled out their questionnaire at home or in the dialysis department. The last laboratory data was obtained from the patients’ data file. Dialysis staff rechecked the written information by the patients to assure correct understanding and to make sure that they completed the questionnaire.

**Table 1.** Internal Consistency of Reliability (Cronbach Alphas)

	Cronbach’s alpha
**Symptoms**	0.91
**Effects of kidney disease**	0.82
**Burden of kidney disease**	0.65
**Work status**	0.42
**Cognitive function**	0.66
**Quality of social interaction**	0.53
**Sexual function**	0.86
**Sleep**	0.64
**social support**	0.81
**Dialysis staff encouragement**	0.70
**Patient satisfaction**	----
**Physical function**	0.91
**Role physical**	0.73
**Pain**	0.78
**General health**	0.60
**Emotional well being**	0.70
**Role emotional**	0.71
**Social function**	0.41
**Energy/ fatigue**	0.53

### 3.3. Definition

In all HD patients, serum HBsAg, HBS Abs, and HCV Abs [enzyme-linked immunosorbant assay (ELISA)] were routinely checked every 6 months. Patients were considered for chronic HBV infection if HBsAg was positive for more than six months (7). In all HD patients, third generation assay was used to detect HCV infection. Furthermore, serum HCV-RNA (PCR) was examined for anti-HCV-positive patients for confirmation of HCV infection.

### 3.4. Data Analysis

Statistical analyses were performed with SPSS 18.0 for Windows. Clinical, demographic, and HRQOL variables were expressed as means and standard deviations. Categorical variables were measured as frequencies and percentages. One-way analysis of variance (ANOVA) or Kruskall-Wallis tests for skewed data were used to compare continuous variables between more than two groups; the Student’s t-test or the Mann-Whitney test for skewed data was applied for comparisons between two groups. The Chi square test was used to compare categorical variables. Pearson correlation was used to assess the relationship between quality of life and continuous variables (e.g. age, dialysis vintage, Kt/V, and Hb). Following the univariate analysis, all demographic and clinical variables with P ≤ 0.2 were entered as predictor variables in multiple regression models. A P-value of 0.05 or less was considered to indicate statistical significance.

## 4. Results

From a total of 4101 dialysis patients aged between 14 and 91, 2.1% and 1.3% had hepatitis B and hepatitis C infection, respectively. In more than half of all patients (57.4%) and in all 3 groups, male gender was predominant (Table 2). The mean age was 55 ± 16 years with a mean dialysis vintage of 39±40 months. The most common primary known disease was hypertension (30.7%). More than half of the participants were educated (54%) and near 60% were aged less than 60 years old. Mean age for non-HBs non-HCV, HBs Ag positive and HCV Ab positive patients were 55.22 ± 16.29, 49.85 ± 16.62 and 47.82 ± 13.51, respectively, P = 0.001.

**Table 2.** Comparison of Demographic Information Between Groups

Demographic information	Non B non C, No. (%)	HBS positive, No. (%)	HCV positive, No. (%)	P value
**Dialysis vintage**	38.15 ± 39.11	55.91 ± 41.52	85.35 ± 68.05	< 0.001
**Hypertension**	1211 (31.7)	29 (35.4)	22 (23.1)	0.3
**Diabetes **	1026 (26.9)	16 (19.5)	1 (1.9)	0.3
**ADPKD ** **^a^**	154 (4)	3 (3.7)	3 (5.8)	0.3
**SLE ** **^a^**	111 (2.9)	6 (7.3)	0	0.3
**Infection**	205 (5.4)	3 (3.7)	8 (15.4)	0.3
**Others **	442 (11.6)	6 (7.3)	6 (11.5)	0.3
**Unknown**	669 (17.5)	19 (23.2)	12 (23.1)	0.3
**Educational state**				< 0.001
Uneducated	1752 (49.8)	42 (52.5)	13 (27.7)	
Primary school	906 (25.8)	19 (23.8)	13 (27.7)	
High school	773 (22)	18 (22.5)	16 (34)	
University	86 (2.4)	1 (1.3)	5 (10.6)	
**Gender**				0.001
Male	2259 (57)	63 (74.1)	38 (71.7)	
Female	1704 (43)	22 (25.9)	15 (28.3)	
**Age**				0.004
≤ 45	1047 (26.8)	12 (34.3)	22 (42.3)	
46 – 60	1267 (32.4)	14 (40)	18 (34.6)	
> 60	1592 (40.8)	9 (25.7)	12 (23.1)	
**Nationality**				< 0.001
Iranian	3818 (97.2)	76 (89.4)	47 (88.7)	
Non-Iranian	110 (2.8)	9 (10.6)	110 (2.8)	
**Marital state**				< 0.001
Single	408 (10.5)	10 (12.3)	19 (38)	
Married	2876 (74.3)	60 (74.1)	27 (54)	
Widowed/Divorced	586 (15.1)	11 (13.6)	4 (8)	
**Work status**				0.001
Employment	348 (9)	8 (9.6)	13 (25)	
Unemployment	1513 (39.2)	38 (45.8)	22 (42.3)	
Retired	724 (18.8)	19 (22.9)	7 (13.5)	
House worker	1246 (32.3)	17 (20.5)	10 (19.2)	
Student	27 (0.7)	1 (1.2)	0	

^a^Abbreviations: ADPKD, autosomal dominant poly cystic kidney disease; SLE, systemic lupus nephritis

### 4.1. Viral Hepatitis

Patients with HCV infection were younger than those who had no hepatitis B and C (48 ± 13 vs 55 ± 16 years, P = 0.001) and individuals with HBV infection (48 ± 13 vs 50 ± 17 years, P = 0.5). Half of patients in group II were uneducated (52.5%), while majority of patients with HCV Ab positive were educated (72.3%). The prevalence of HBV and HCV infections was significantly higher in Afghani refugee HD patients compared to the Iranian population (7.2% vs 1.9% P = 0.001 and 4.8% vs 1.2%, P = 0.004 respectively). Male gender was predominant for HBV and HCV infections (male to female ratio=2.1:1 and 1.8:1, respectively). It is important to note that almost all KDQOL items for viral hepatitis patients had equivalent or better scores than those without viral hepatitis (Table 3). Although burden of kidney disease had a lower score in HBsAg positive patients when compared to HCV Ab positive patients and negative HBV and HCV patients, this was not statistically significant (20.1 ± 18.5 vs 25.58 ± 22.2 and 22.59 ± 18.7, P = 0.3). The other demographic data and KDQOL results for HBS positive, HCV positive and non-infected viral hepatitis patients are summarized in Table 2.

**Table 3.** Comparison of KDQOL Items Between Groups

	No Hepatitis B and C	HBS Positive	HCV Positive	P Value
**Symptoms**	66.83 ± 19.90	76.61 ± 15.69	70.49 ± 18.48	0.006
**Effects of kidney disease**	50.80 ± 20.68	54.65 ± 15.61	52.58 ± 27.44	0.4
**Burden of kidney disease**	22.59 ± 18.76	20.1 ± 18.58	25.58 ± 22.27	0.3
**Work status **	24.41 ± 33.94	24.32 ± 36.55	29.94 ± 38.52	0.5
**Cognitive function**	65.54 ± 20.29	68.19 ± 19.27	67.92 ± 24.93	0.5
**Quality of social interaction**	66.46 ± 19.52	68.79 ± 18.9	68.55 ± 20.28	0.5
**Sexual function**	62.66 ± 30.07	61.36 ± 27.64	73.61 ± 31.52	0.5
**Sleep**	56.31 ± 18.83	56.75 ± 18.63	54.29 ± 21.49	0.7
**Social support**	72.39 ± 27.55	82.86 ± 25.66	77.88 ± 25.71	0.02
**Dialysis staff encouragement**	83.09 ± 19.91	92.36 ± 15.31	86.08 ± 19.40	0.01
**Patient satisfaction**	72.95 ± 22.40	78.82 ± 22.09	73.27 ± 23.87	0.2
**Kidney disease component summary (KDCS)**	58.16 ± 11.33	61.93 ± 10.38	60.64 ± 14.34	0.04
**Physical function**	38.48 ± 28.09	48.65 ± 30.73	50.75 ± 31.24	0.001
**Role physical**	25.41 ± 32.37	35.13 ± 36.06	28.3 ± 35.36	0.1
**Pain**	53.83 ± 24.45	69.18 ± 23.47	59.43 ± 28.82	< 0.001
**General health**	41.88 ± 19.00	45.74 ± 15.73	45.77 ± 25.4	0.1
**Physical component summary (PCS)**	39.89 ± 19.15	49.68 ± 20.64	46.06 ± 23.56	0.001
**Emotional well being**	54.41 ± 16.86	60.27 ± 18.40	54.96 ± 24.34	0.1
**Role emotional**	34.02 ± 37.76	40.54 ± 42.4	39.74 ± 40.71	0.3
**Social function**	53.80 ± 21.56	59.79 ± 20.86	63.44 ± 26.49	0.001
**Energy/ fatigue**	45.97 ± 18.50	49.81 ± 21.88	45.34 ± 24.08	0.4
**Mental component summary (MCS)**	47.07 ± 17.33	52.20 ± 20.81	50.78 ± 22.68	0.06
**SF-36**	43.48 ± 16.87	50.94 ± 20.02	48.42 ± 21.48	0.003
**SF-36+KDCS**	50.82 ± 12.79	56.43 ± 13.71	54.53 ± 16.81	0.004
**Dialysis duration**	38.15 ± 39.11	55.91 ± 41.52	85.35 ± 68.05	< 0.001
**Age**	55.22 ± 16.29	49.85 ± 16.62	47.82 ± 13.51	0.001

In HBsAg positive patients, duration of dialysis had no significant correlation with any of the KDQOL items. But increasing age had a negative significant effect on symptoms (P = 0.01), cognitive function (P = 0.007), quality of social interaction (P = 0.001), patient satisfaction (P = 0.01), pain (P = 0.02) and social function (P = 0.02). In HCV Ab positive patients, we found a positive correlation between duration of dialysis, work status (P = 0.02) and pain (P = 0.03), however, age had a negative correlation with role physical function (P = 0.004), role physical (P = 0.006), pain (P < 0.001), general health (P = 0.02), PCS (P < 0.001), emotional wellbeing (P = 0.04), energy fatigue (P = 0.03), role emotion (P = 0.04), social function (P = 0.02) and MCS (P = 0.007). In logistic regression after adjustment for age, sex, educational level, marital state, dialysis vintage, HBs Ag positivity and HCV Ab positivity; only age (P < 0.001) and educational level (P = 0.015) had a negative impact on quality of life (total SF- 36 and KDCS).

## 5. Discussion

In the current study, we observed that the prevalence of HBS antigenemia and HCV Ab positivity among HD patients decreased from 2.6% and 4.5% in 2006 (7) to 2.1% and 1.3% in 2011, respectively. In this survey, we demonstrated that most of the KDQOL scores were higher in HD patients with viral hepatitis; especially PCS had significantly better scores for the 3 major components of the KDQOL questionnaire KDCS.

### 5.1. Incidence

There is a wide variation in the prevalence of HBV and HCV infections among different HD units and countries as shown by previous studies (7, 9, 18). The mean prevalence of HCV infection in different dialysis centers is approximately 13.5% and varies among different countries from 2.6% to 22.9% (9), while the prevalence of HBV infection within dialysis units in developing countries is 2% – 20% (9). Although in a previous study (9) HBV infection was less prevalent than HCV infection in HD units, we showed that HCV infection had a lower prevalence than HBS Ag enemia in most of the Iranian dialysis centers. Despite the fact that we did not isolate HCV positive patients in dialysis units, lower rate of HCV infection may be due to a successful strategy for viral hepatitis prevention in HD units such as screening of all HD patients to detect HCV Ab using third generation. There is a limited data about occult HCV infection in HD patients (19-22). Barril and colleagues detected genomic HCV RNA in peripheral blood mononuclear cells from 45% of long-standing HD individuals who had an unexplained high aminotransferases and had repeated anti-HCV antibody and serum HCV RNA negative. In addition, HCV-related liver disease in ESRD patients is characterized by spuriously low levels of liver enzyme, even in the presence of active infection. Moreover, some researchers have shown that occult HCV infection can be found following spontaneous or treatment-induced clearance of serum HCV-RNA (19). Thus, using only third generation assay could not exactly detect HCV infection and we used only HCV-RNA detection in anti HCV Ab positive; hence, our reported prevalence of HCV infection may be underestimated.

### 5.2. HRQOL

Chronic infections such as chronic viral hepatitis can impair HRQOL by affecting memory, neurocognitive function, concentration, attention and sexual function (5, 23). Conversely in this study, symptom scores were significantly better in HD patients with viral hepatitis. According to some studies, most patients infected with HCV and HBV infections are asymptomatic (1, 3) because the natural histories of these infections extend over decades rather than years and adverse consequences of chronic HBV or HCV infections may not be obvious in patients followed for a short period of time (3). Thus it is possible that the symptoms in our HD patients were not severe enough to have a signiﬁcant effect on their quality of life. The total scores for KDCS and PCS were also significantly better in these groups of patients. Some explanations are as follows:

1) As we know HRQOL is a multidimensional concept that includes physical, mental, pysico-social, and spiritual aspects of health. In addition, many biological and non-biological factors such as hemoglobin levels, mineral and inflammatory biomarkers, Kt/V, patient’s culture, religious behaviors, and ethics, may play an important role in HRQOL because they can affect patients’ perceptions and expectations regarding quality of life (24). Therefore, the KDQOL questionnaire has defects in some human aspects of life (25) and cannot assess all aspects of life. Thus the instrument used in the present study was not sensitive enough to identify mild cognitive impairments. Moreover, we also have limitations in this survey, because we didn’t consider any of the biological factors, which seriously contribute to HRQOL and as a result unexpectedly many contributing factors were ignored.

2) On the other hand, distribution of viral hepatitis varies in different providence of Iran. Thus we may have a selection bias in this study, but a large number of HD patients were included; therefore, the results may be representative for all Iranian HD patients.

3) It seems that chronic hepatitis in patients, especially HCV infection, has small but signiﬁcant effects on higher neurological functioning, however the impact of these changes on physical and mental well being is poorly understood and presumably links to both the degree of the change and the personal adaptation that, in some patients, may lessen the effects of infection on HRQOL (23).

4) The HRQOL was considerably diminished in HD patients, but less so in the group that was educated about their nutrition (8). Also, according to previous studies, male and employed patients have better HRQOL (15, 26, 27). Interestingly, our patients with viral hepatitis were significantly younger, of the male gender, more employed and had higher education. Subsequently they were more likely to have better HRQOL scores.

5) Although there are some reports about quality of life in ESRD patients and chronic HCV infected patients, there is little reported data about HRQOL in treated and untreated HD patients with chronic HCV infection (28). Foster and colleagues in 2009 showed that sustained viral response can improve HRQOL. For the majority of patients, viral clearance produces improvements in both HRQOL and long-term prognosis (23). We also had another limitation that is we did not exclude treated patients or HBV carriers who have better HRQOL than patients with active hepatitis (6).

### 
